# The contradictory effects of coffee intake on periodontal health: a systematic review

**DOI:** 10.12688/f1000research.124547.2

**Published:** 2022-10-17

**Authors:** Taufan Bramantoro, Amalia Zulfiana, Muhammad Subhan Amir, Wahyuning Ratih Irmalia, Nor Azlida Mohd Nor, Alexander Patera Nugraha, Agung Krismariono

**Affiliations:** 1 Department of Dental Public Health, Faculty of Dental Medicine Universitas Airlangga, Surabaya, East Java, 60132, Indonesia; 2 Department of Oral and Maxillofacial Surgery, Faculty of Dental Medicine Universitas Airlangga, Surabaya, East Java, 60132, Indonesia; 3 Indonesian Health Innovation and Collaboration Institute, Surabaya, East Java, 60176, Indonesia; 4 Department of Community Oral Health and Clinical Prevention, Faculty of Dentistry University of Malaya, Kuala Lumpur, 50603, Malaysia; 5 Department of Orthodontics, Faculty of Dental Medicine Universitas Airlangga, Surabaya, East Java, 60132, Indonesia; 6 Department of Periodontology, Faculty of Dental Medicine Universitas Airlangga, Surabaya, East Java, 60132, Indonesia

**Keywords:** bone loss, caffeine, coffee, periodontal health, periodontitis, tooth loss

## Abstract

**Background:** Drinking coffee is known to have both positive and negative aftermath on periodontal health. The current study is aiming to systematically review the impact of coffee consumption on periodontal health status. **Methods:** An article search was carried out in two electronic databases (PUBMED and Web of Sciences). The assessment of the included articles were conducted using Joanna Briggs Institute (JBI) critical appraisal tool. Data were analyzed qualitatively. **Result: **A total of 10 articles were included in this study. Most (5) of the studies discovered a negative correlation between coffee intake and periodontal health, while 4 other studies found the protective effect of daily coffee consumption against alveolar bone loss. Last, only one study found that coffee intake did not relate with periodontitis. **Conclusion: **The effect of coffee consumption on periodontal health was fragmented since coffee has complex components that may give either beneficial effects or negative impact on periodontal health.

## Introduction

Coffee has been considered as one of the most consumed beverages among adults worldwide. ^1^ Reported by International Coffee Organization, the world consumption of coffee increased by 1% from 2017 to 2021, with the highest increase found in Africa and the largest consumption reported in Europe. The similar trend also happened on the coffee production by the coffee-exporting-countries, showing an increase of 6.3% in a year since 2019. ^2^

There are several reasons for people decided consuming coffee or not. For coffee drinkers, one of the leading motives reported are due to its health benefits, taste and pleasure. ^3^ In terms of health, a systematic review reported drinking coffee is associated with the lower risk of diabetes. ^4^ Other protective effects of coffee intake had been reported in epidemiological studies; giving therapeutic effect for cardiovascular diseases, Alzheimer’s disease, gastritis, and other chronic diseases. ^5^ Meanwhile, some other people may still avoid drinking coffee due to their belief for its adverse effects, such as anxiety and insomnia. ^3^^,^^6^ Thus, aside from giving several health benefits, daily coffee intake in adequate dose might also had some negative impacts. ^3^

The contradictory effects of daily coffee intake are also found in oral health fields. Many studies reported the protective effects of coffee consumption on periodontal health, while others discovered its drawbacks. The antioxidant and anti-inflammatory effects of caffeine contained in coffee might lead to beneficial results for periodontal diseases. In contrary, a study indicated that the daily coffee consumption may delay the bone repair after tooth extraction. ^7^ Thus, we are aiming to systematically review the impact of coffee consumption on periodontal health status.

## Method

### Search strategy

We used two electronic databases (PubMed and Web of Science) as our tools to search the potential literatures. The literature search through databases was performed on 6 April 2022 until 14 April 2022. We constructed the keywords for each database. For PubMed, we used the following keywords; ((((((coffee [MeSH]) OR (caffeine [MeSH])) OR (“coffee intake”)) OR (“coffee consumption”)) OR (“caffeine intake”)) OR (“caffeine consumption”)) AND ((((mouth [MeSH]) OR (tooth [MeSH])) OR (((“oral health” [MeSH]) OR (“periodontal health”)) OR (“periodontal index” [MeSH]))) OR (((“tooth loss”[MeSH]) OR (periodontitis [MeSH])) OR (periodontal disease [MeSH]))). Meanwhile for Web of Science, the keywords were (((((ALL=(coffee)) OR ALL=(“coffee intake”)) OR ALL=(“coffee consumption”)) OR ALL=(“caffeine intake”)) OR ALL=(“caffeine consumption”)) OR ALL=(caffeine) AND (((((((ALL=(mouth)) OR ALL=(tooth)) OR ALL=(“oral health”)) OR ALL=(“periodontal index”)) OR ALL=(“periodontal health”)) OR ALL=(“periodontal disease”)) OR ALL=(periodontitis)) OR ALL=(“tooth loss”). During the literature search, no time limit was set.

### Inclusion criteria

All types of experimental and observational studies in English language were included. No duplicate studies and no treatment or clinical trial studies included. Study subjects include adults without any gender and age restrictions, and any other objects of *in vivo* and *in vitro* studies. Study factor or exposure included in the studies were coffee or caffeine intake in a daily basis, any other interventions using caffeine or coffee. Outcome of studies included were oral health status, periodontal health status, periodontal diseases, tooth loss and any other assessment of periodontal conditions.

### Study selection, data extraction, and quality assessment

**Figure 1.**
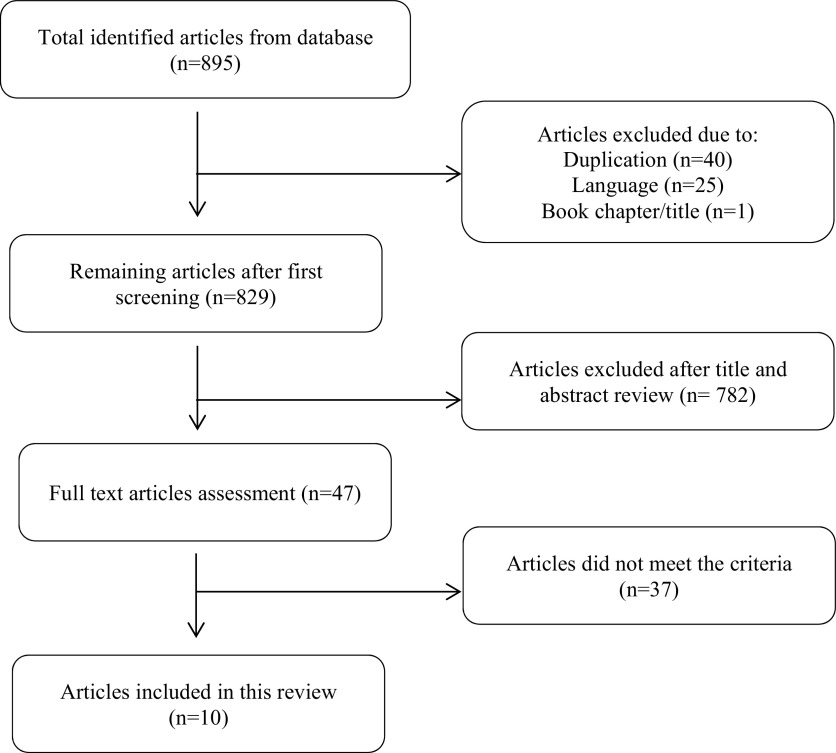
The flow diagram of the study selection. ^26^

The aforementioned keywords gave a total number of 895 articles, consisting of 243 and 652 articles from PubMed and Web of Science respectively. We excluded one literature as it was a book chapter. After excluding duplicates and languages, we had 829 potential articles to be reviewed. After conducting title and abstract reading, we had remaining 47 studies. Two researchers reviewed the full text of those studies and finally selected ten of them which met the inclusion criteria. Critical appraisal had been done by two reviewers separately using JBI Critical Appraisal tools. The flow diagram of the selection of study is shown in Figure 1.

## Results

### Characteristics of the articles

The included studies in this review were originally from Asia, America, Europe, and Africa. Out of ten studies, three were from Korea, two were from Japan, another two were from America, and the remaining three were from each of Brazil, Germany, and Egypt. The year of publication ranged from 1999 to 2022. Half of the included studies used cross-sectional study design, three studies were randomized controlled trial, one study used cohort study design, and another one used quasi-experimental study design. This review including studies with various subject, six studies observing the effect of coffee intake on human, ^8^^–^^13^ three studies exploring the cellular changes using rats as the subject, ^14^^–^^16^ and one *in vitro* study observing the coffee effect using UMR106-01 rat osteoblast-like cells. ^17^ The summary of the included study were tabulated based on the results, whether coffee intake have positive ( Table 1) or negative effect ( Table 2) on periodontal health.

**Table 1.** The summary of studies revealed positive effect of coffee on periodontal health. ^27^

Author, Year	Coffee intake (exposure)	Oral health assessment (outcome)	Setting	Findings	Conclusions
Ng, *et al.,* 2013	Coffee intake	Moderate-to-severe periodontal disease	Community, participants in the US Department of Veterans Affairs Dental Longitudinal Study (DLS)	Higher coffee intake lead to significant decrease in the number of teeth with alveolar bone loss. There is no evidence showing that the consumption of coffee negatively impact the periodontal health.	There is a beneficial association between of coffee intake and periodontal health, yet its extent was small and may not cause any clinical significance.
Kobayashi, *et al.*, 2020	0.62% or 1.36% coffee components contained in food	Alveolar bone loss	In vivo, Male Fischer 344 rats	The nuclear levels of Nrf2 were higher in the 1.36% coffee group than in the control group. Level of total antioxidant capacity were higher in the 1.36% coffee group.	In rats, the long-term consumption of 1.36% coffee might prevent age-related oxidative stress in periodontal tissue and alveolar bone loss, using the up-regulation of Nrf2 signaling pathway. The antioxidant effects of coffee were found greater in the highest percentage of coffee in a diet.
Kim, YR and Nam, SH., 2019	Coffee only, coffee with cream, coffee with syrup, coffee with cream + syrup	The orevalence of periodontal disease, the community periodontal index (CPI)	Community, using data from the Korean National Health and Nutrition Survey (KNHANES)	Consuming coffee with cream could lower the prevalence of periodontal disease (0.83-times lower than drinking black coffee).	Drinking coffee with cream might give beneficial health effects to South Korean adults as it might improve oral health, reducing the prevalence of periodontal disease.
Machida, *et al.*, 2014	Coffee intake frequency (≥ 1 cup/day and <1 cup/day)	Pocket Depth (PD), Clinical Attachment Level (CAL), Bleeding on Probing (BOP) Periodontitis severity (severe, moderate, mild)	Hospital, chronic periodontitis patients at Okayama University Hospital	The ≥1 cup/day group had less severity of periodontitis compared to the group consuming <1 cup/day.	A routine coffee intake was negatively associated with severe periodontitis in the maintenance phase of periodontal therapy.
Hong, *et al.*, 2021	Regular coffee/green tea/soft drink intake (none, mild, heavy drinkers)	The presence of periodontitis	Community, epidemiological data from the Korean Genome and Epidemiology Study	No statistical significance between coffee intake and periodontitis.	There is no association between the regular coffee intake and periodontitis. Low frequency of soft drink consumption might be related to periodontitis.

**Table 2.** The summary of studies discovered the drawbacks of coffee consumption on periodontal health. ^27^

Author, Year	Coffee intake (exposure)	Oral health assessment (outcome)	Setting	Findings	Conclusions
Abbas, *et al.*, 2018	Green coffee bean extract (GC group); Agiolax ^®^ (Ag group)	Histological, histomorphometrical, and western blotting examination on alveolar bones	*In vivo* (Wistar albino rats)	A significant decrease of the bone area (%) and serum calcium level in the GC groups. An increase of calcium level in urine in the two experimental groups. RANKL expression was increased and decreasing tissue calcium level was only found in the GC group.	The daily intake of green coffee bean extract might have strong association with alveolar bone loss. The GC group showed more damaging effects such as decreased Ca Level in tissue, reduced the bone area percentage, and more RANKL expression.
Bezerra, JP., *et al.*, 2008	Caffeine intake (10 mg/100g body weight/day for 56 days)	Histometrical examination of periodontal ligament, bone loss in the first molar furcation	*In vivo*, using Wistar rats	The caffeine group showed a larger area of bone loss on the ligated tooth	The high dose of caffeine consumption might accelerate the progression of ligature-induced periodontitis.
Han, K., *et al.*, 2016	Consumption of coffee	Periodontal index	Community data from the Korea National Health and Nutrition Examination Survey	The prevalence of coffee consumption was significantly greater in the male periodontitis patients.	In Korean male adults, coffee consumption might be considered as an independent risk indicator of periodontitis.
Kiyoura, YK., *et al.*, 1999	Caffeine (0.1mM and 1mM)	The presence of prostaglandin E2/PGE2 (0.1μg/ml and 1μg/ml)	*In vitro*, using UMR106-01 rat osteoblast-like cells	After at least 72 or 96 hours in the medium, the combination of caffeine and PGE2 can give inhibitory effects to cell proliferation.	Long-term caffeine consumption could be a potential risk factor for periodontitis, deteriorating the pathological condition of the periodontitis patient.
Struppek, *et al.*, 2022	Strong (≥ 7 or more cups/day); moderate (3-6 cups/day); low (0-2 cups/day) coffee consumption	Periodontitis (mild, moderate, or severe)	Community, general population of Hamburg	The participants presented either none/mild (n = 1,453, 39.6% men, 2.4% strong coffee consumers), moderate (n = 3,580, 49.3% men, 3.3% strong coffee drinkers), or severe (n = 1,176, 60.9% men, 5.0% strong coffee consumers) periodontitis.	In a northern German population, there is a significant positive association between strong coffee consumption and periodontitis.

### Methodological quality

*Human studies*

Among six studies, two studies used data from national survey, and choosing the eligible participants based on some criteria. One study used data from dental longitudinal studies, one study used data from Genome and Epidemiology Study, one used general population, and one other study selected chronic periodontitis patients who had received initial treatment as the participants of the study. All of the human studies used questionnaire to assess the dose of daily coffee consumption (all six studies), ^8^^–^^13^ one study also used Cornell Medical Index (CMI) to gain coffee consumption in addition to questionnaire. ^9^ Two studies admitted that validity and reliability test of the questionnaire were not performed sufficiently, therefore, further research is required. ^11^^,^^12^

The assessment of periodontal health was mostly performed using various reliable ways, however, one study only ask whether the participants ever diagnosed with periodontitis without further clinical examination. ^11^ The clinical oral examination performed to assess periodontal health including scoring method according to American Association of Periodontology by probing pocket depth and clinical attachment level, ^12^ using Community Periodontal Index (CPI), ^8^^,^^10^ radiographic examination to measure alveolar bone loss, measurement of probing depth, ^9^^,^^13^ bleeding on probing, ^9^^,^^13^ gingival recession, ^13^ and examining supragingival calculus, ^9^^,^^13^ and plaque index score. ^13^ Aside from periodontal health, caries measurement using DMFT index was also performed. ^13^

In terms of bias, some studies also observed several confounding factors, such as Body Mass Index (BMI) ^9^^,^^11^^,^^13^ and other nutritional intake, ^11^ smoking and alcohol habit, ^9^^,^^11^^–^^13^ medical history such as diabetes, ^8^^,^^9^^,^^12^^,^^13^ cholesterol level ^8^ and coronary heart diseases, ^12^^,^^13^ blood sample analysis, ^8^ and other socio-demographic factors. ^10^^,^^12^

*Animal studies*

The animal studies were performed with various designs. One study induced periodontitis on rats by means of ligature placement, then made comparisons between groups with and without caffeine ingestion. ^15^ The two other studies observed the effect of giving green coffee bean extract ^14^ and powdered coffee ^16^ on periodontal health of the rats. Histological examinations were performed to assess the effect, by observing bone structure and volume, ^14^^–^^16^ RANKL expression, ^15^ measuring serum oxidative stress and antioxidant capacity, 8-OHdG and Nrf2 positive cells, and gene expression analysis. ^16^

*In vitro study*

The only one *in vitro* study was observing any possible interaction between Prostaglandin E2 (PGE2) and caffeine using UMR106-01 rat osteoblast-like cells. ^17^

## Discussion

### Key findings

*The favorable effects of coffee intake on periodontal health*

The minority of the included studies managed to reveal the benefits of coffee intake on periodontal health. Studies found that coffee intake might be beneficial against periodontal disease. ^9^^,^^10^ Another study on patient with periodontitis during maintenance phase of therapy also showed those who drank more than one cup of coffee in a day had lower prevalence of severe periodontitis. However, no such significant difference found on the prevalence of moderate periodontitis. ^12^ An *in vivo* study using revealed that consuming coffee had a protective effect against periodontal diseases. The result showed a lower ratio of 8-OHdG-positive cells in the group with the highest dose of coffee intake, compared to those in the control and other treatment group with lower dose of coffee. The study also identified that coffee intake improve the antioxidant activity in periodontal tissue by upregulating Nrf2 signaling pathway. ^16^ However, Hong *et al* (2021) revealed that there is no significant association between coffee intakes and periodontitis as the adjusted ratio were not statistically significant. ^11^

*The drawbacks of coffee consumption on periodontal health*

Five studies found out that consuming coffee might also have negative impacts on periodontal tissue, especially the alveolar bone. An animal study comparing the effect of consuming two different dietary supplement (green coffee extract/GC and Agiolax ^®^/Ag) on alveolar bone loss. Both supplements showing deleterious effect towards periodontal health, however, GC groups showed greater alveolar bone loss compared to Ag groups. ^14^ Another study on periodontitis-induced rats by placing a cotton ligature also showing that caffeine ingestion causing larger area of bone loss, even though without ligature placement, caffeine alone was unable to induce alveolar bone loss. ^15^ The only in-vitro study in this review investigated the interaction between caffeine and prostaglandin E2 (PGE2) on rats’ osteoblast-like cells. It showed significant inhibition of cell proliferation when both caffeine and PGE2 were incubated together and the inhibition were stronger as the dose higher. ^17^ Lastly, two human studies also indicated the destructive effect of coffee on periodontal tissue. ^8^^,^^13^

### Strength and limitation of the review

Interestingly, the current review covers several study designs and settings, taking humans, animals, and culture medium as their objects of study. However, some studies did not state the possible confounding factors and ways to deal with it. ^8^^–^^10^

### Significance of the findings and possible mechanism

Periodontitis is an inflammatory disease that mainly caused by microorganisms that present as the destruction of teeth supporting tissue. Periodontitis is also associated with the presence of reactive oxygen species (ROS) as a result of hyperactivity of peripheral blood neutrophils. Its hyperactivity may occur as a reaction of host-immune reaction to the inflammation of periodontitis. Not only neutrophils, the infection of bacteria producing ROS might also be contributed to the oxidative stress in periodontitis, leading to the progression of alveolar bone loss.

Coffee is considered as one of the most antioxidant-rich beverages as it has various components that were found to have antioxidant and anti-inflammatory properties; caffeine, chlorogenic acid, and caffeic acid. Due to the presence of such effects, there might be protective impacts of coffee in periodontal diseases. ^8^^,^^10^ A study suggest that phenolic content in coffee have strong antioxidant properties, thus might reduce oxidative stress due to bacterial activity. In addition, the recent replacement of cream with skimmed milk powder in the coffee mix is thought to be helpful in preventing osteoporosis, thus can also be protective against periodontal bone loss. ^10^^,^^18^ Milk belongs to dairy products, with calcium and casein content that is helpful in bone and tooth mineral preservation. ^18^ Other explanation of the coffee protective effect against alveolar bone loss is also presented by Ng *et al.* It was stated that caffeine has immunomodulatory actions by inhibiting cyclic adenosine monophosphatase (cAMP)-phospodiesterase, thus increasing the concentration of intracellular cAMP. ^9^ Another supporting result was also presented by Machida et al that observed the effect of consuming coffee during the maintenance phase of periodontal therapy. ^12^ Besides, chlorogenic acid contents in coffee also known to act as antimicrobial agent by reducing the protease activity of *Porphyromonas gingivalis.* Those are bacteria that commonly found in plaque biofilm that plays role in the periodontal disease progression. ^11^^,^^19^

A cellular molecular observation also found that chlorogenic acid contained in coffee can significantly decrease malondialdehyde, which is the result of lipid peroxidation degradation, and increase catalase, superoxide dismutase, and glutathione. Thus, reducing oxidative stress. However, the study was focus on the anti-aging effect of coffee intake toward oxidative stress on periodontal tissues, without considering the normal flora. Thus, the effect on periodontal disease might be slightly different. ^16^

The similar findings have been shown through gene expression analysis. Some genes that were having a role in the antioxidant effect were highly expressed in the highest percentage of coffee group; glutamate cysteine ligase modifier subunit, ferritin, and hypoxanthine phosphoribosyltransferase 1 genes. ^16^ Glutamate cysteine ligase (modifier subunit) has a role to maintain the synthesis of glutathione, one of the largest intracellular antioxidants. Meanwhile, ferritin has a protective role against iron-dependent oxidative stress. The cellular defense against oxidative stress may occur through the activation of Nrf2 signaling pathways which controls the genes that involved in the elimination of reactive oxidants by increasing the cellular antioxidant capacity. ^20^ This notion is consistent with the study result showing the more frequent of Nrf2 nuclear translocation in the highest percentage of coffee group. ^16^ It is indicated that the continuous intake of coffee lead to some increase in the antioxidant capacity of the periodontal tissue, decreasing age-related oxidative stress in the tissues. The systemic increase of antioxidant activities may contribute to a decrease in oxidative damage at the local level. ^21^ Hence, the systemic approaches including dietary habits may also provide promising benefits in the treatment of periodontal diseases. ^12^

In contrast, many studies reported the detrimental effects of coffee intake on periodontal disease. ^8^^,^^13^^–^^15^^,^^17^ Hypotheses explaining the association might focus on the caffeine as the major content in coffee. ^8^^,^^14^ Caffeine is reported to affect calcium metabolism and evoking bone mineral density. Thus, long-term caffeine intake denotes one of the risk factors in the advancement of periodontitis pathology. Another study revealed that caffeine may increase bone loss and alter bone healing following tooth extraction. ^8^ There are several pharmacological and cellular activities of caffeine on bone metabolism related to osteoblast proliferation and calcium metabolism. ^8^^,^^14^^,^^15^ Caffeine is known to be able to inhibit the osteoblast-like cells proliferation, also have negative impacts towards the viability of osteoblast, leading to the increase of apoptosis rate of the cells. ^15^ The negative impacts towards these cells are due to the caffeine ability to disrupt the mitochondria of osteoblast and osteocyte in vivo. ^17^ Caffeine can also increase the expression of the receptor activator of NF-κB ligand (RANKL), as the result of low calcium levels in blood. The low level of calcium will be responded by the increase secretion of parathyroid hormone, and stimulate the osteoclast to increase calcium blood levels through expressing RANKL. ^14^ Alveolar bone loss in periodontitis is mediated by tumor necrosis factor (TNF)-α, interleukin (IL)-6, RANKL, and prostaglandin E2 (PGE2) as the response to a pathogens. A combination of PGE2 and caffeine in blood strongly inhibit osteoblast proliferation. ^15^^,^^17^ This might be the reason behind the absence of coffee consumption effect toward healthy periodontal tissue. The contradictory effects of coffee consumption might be caused by several factors. First of all, the roast degree can alter the amount of chlorogenic acid content in the coffee, thus might also affect its anti-oxidant, anti-bacterial and anti-inflammatory activity. ^19^ Secondly, the caffeine content in each coffee consumed may vary, and the last possible causes is the additives content in coffee (milk, sugar, cream) consumed might also contribute to different effects of coffee. ^22^ In addition, the effects of coffee on health work in a dose-dependent manner. The high dose of caffeine administered daily in an *in vivo* study that causes bone destruction was equivalent to 1,360mg/70kg ^0.75^ in humans. ^15^ This dosage was equivalent to 16 cups of coffee per day. Meanwhile, the suggested daily intake of coffee that is considered to be safe is around four-five cups per day.

The maximum plasma concentration of caffeine is reached 15-120 minutes after ingestion. While the threshold of caffeine toxicity was recorded around 400 mg/day in healthy adult, 100 mg/day in healthy adolescent, and 2.5 mg/kg/day in healthy children aged less than 12. Meanwhile, during pregnancy, the suggested dose of caffeine consumption is less than 200 mg/day. The average dose of caffeine contained in coffee varies between 30–175 mg/cup, moderate coffee intake around 2–3 cups/day is considered safe. ^23^^,^^24^

## Conclusion

The effect of coffee consumption on periodontal health was fragmented since coffee has complex components that may give either beneficial effects or negative impact on periodontal health. Drinking coffee in routine and in a proper dosage may give benefits on periodontal health, but it also potentially has detrimental effects if excessive dosage consumed.

### Strengths, limitations, and recommendations

As we did limit the language to be English only, many studies written in non-English were not covered in this review which may lead to language bias. The various designs used in the selected studies gave a wide range of results and it is not feasible to quantitatively summarize the selected studies. Besides, the measurement of periodontal health as the study outcome is various, giving a challenge to meaningfully compare among one and another. Moreover, the majority of studies were a cross-sectional study which cannot answer the causality of the findings. In future studies, it will be necessary to observe the potential antioxidant effects on periodontal tissue by measuring the antioxidant marker on gingival crevicular fluid (GCF) sample.

## Data availability

All data underlying the results are available as part of the article and no additional source data are required.

## Reporting guidelines

Figshare: PRISMA_2020_checklist. The contradictory effects of coffee intake on periodontal health- a systematic review. DOI: https://doi.org/10.6084/m9.figshare.20412261.v1. ^25^

Figshare: PRISMA Flowchart. DOI: https://doi.org/10.6084/m9.figshare.20412252. ^26^

Figshare: Table - The contradictory effects of coffee intake on periodontal health- a systematic review. DOI: https://doi.org/10.6084/m9.figshare.20412270.v1. ^27^

Data are available under the terms of the Creative Commons Attribution 4.0 International license (CC-BY 4.0).
