# Supplementary material for: The contradictory effects of coffee intake on periodontal health: a systematic review of experimental and observational studies
Source: F1000Res. 2022 Nov 7;11:924. Originally published 2022 Aug 11. [Version 3] doi: 10.12688/f1000research.124547.3 (PMC9582577; doi:10.12688/f1000research.124547.3)
Supplement: Peer review history

# Peer review: Reviewer response for version 1

**DOI:** 10.5256/f1000research.136751.r150488

**Published:** 2022-10-19

**Authors:** Mohammed Aljunaid

**Affiliations:** 1 Faculty of Dental Medicine, Alsaeed University, Taiz, Yemen

Thank you very much for giving me the opportunity to review this article, I have completed my evaluation and after reading this Systematic Review carefully I am of the opinion that this manuscript should be considered for indexing.

 The idea is very well-conceived and presented clearly, objectives are well organized, and results are completely justifying the hypothesis.

 In general, this manuscript provides sound information and a good contribution in the field dentistry, the study has investigated the systematic review of coffee consumption's impact on periodontal health status.

 Abstract:

 The abstract seems highly well. Methods, results, and conclusions are correlated to the study and are understandable.

 Introduction:

 The literature was clearly stated for publication, but the authors should improve the aim of study.

 Methods:

 Methods are comprehensive, and I do believe they cover the idea.

 Results:

 The results could cover the objectives.

Are the rationale for, and objectives of, the Systematic Review clearly stated?

Yes

Is the statistical analysis and its interpretation appropriate?

Yes

Are sufficient details of the methods and analysis provided to allow replication by others?

Yes

Are the conclusions drawn adequately supported by the results presented in the review?

Yes

Reviewer Expertise:

Oral, Dental Medicine and Periodontology

I confirm that I have read this submission and believe that I have an appropriate level of expertise to confirm that it is of an acceptable scientific standard.

# Response

**Published:** 2022-10-21

**Authors:** Taufan Bramantoro

**Affiliations:** Airlangga University, Indonesia

Thank you very much for the advice.

 We have rewritten our aim to be more specific. Thank you.

# Peer review: Reviewer response for version 1

**DOI:** 10.5256/f1000research.136751.r152682

**Published:** 2022-10-11

**Authors:** Ananto Ali Alhasyimi

**Affiliations:** 1 Department of Orthodontics, Faculty of Dentistry, Gadjah Mada University (UGM), Yogyakarta, Indonesia

The authors of this study have investigated the systematic review of coffee consumption impact on periodontal health status. The authors of the study came to the conclusion, based on the findings of the research, that the effect of coffee consumption on periodontal health was fragmented due to the complex components that coffee contains, each of which has the potential to either give positive effects or have a negative impact on periodontal health. The findings have a number of intriguing implications, and the authors deserve praise for carrying out this research.

 Nonetheless, the authors need to resolve some minor difficulties such as the following:

**General**

 The manuscript's content is relevant to the journal's scope and presents novel findings.

 The study results are significant enough to be worth reading about and have a high impact on Dentistry field

 The title explains clearly what the manuscript is about and draws attention to the significance of the research findings.

 Introduction was well-written, indicate a gap, raise a research question. The author(s) carefully explain the goal of the study and announce the current research, emphasizing what is new and why it matters while also providing up-to-date references.

**Methods**

 Reviewers should take care to minimize potential for bias and weed out unimportant or poorly conducted studies. The authors have followed the procedures for conducting a good systematic review, which include things like coming up with a good clinical question to answer, creating a protocol (with inclusion and exclusion criteria), conducting a thorough and extensive literature search, and screening the abstracts of the studies found in the search, before moving on to reading the selected full texts (PRISMA).

**Results**

 Clear, accurate, and well-presented results that are in line with the methodologies used are presented.

 Discussion and conclusion. The Author(s) logically explained the findings and compared the findings with current findings in the research field. The implications of the findings for future research and potential applications were clearly mentioned. Strengths, limitations, and recommendations were also mentioned detailly.

 Author(s) writes the conclusion: “The effect of coffee consumption on periodontal health was fragmented since coffee has complex components that may give either beneficial effects or negative impact on periodontal health. Drinking coffee in routine and in a proper dosage may give benefits on periodontal health, but it also potentially has detrimental effects if excessive dosage consumed.” Maybe you can add the optimum dose and a safe/proper dose coffee consumption daily.

**References**

 Needs improvement. Some of abbreviations journal were not write properly. Ref no 2 is not accurate. Ref 18 & 18 has not mentioned the name of journal.

Are the rationale for, and objectives of, the Systematic Review clearly stated?

Yes

Is the statistical analysis and its interpretation appropriate?

Yes

Are sufficient details of the methods and analysis provided to allow replication by others?

Yes

Are the conclusions drawn adequately supported by the results presented in the review?

Partly

Reviewer Expertise:

Orthodontics, Biomechanics, Local material

I confirm that I have read this submission and believe that I have an appropriate level of expertise to confirm that it is of an acceptable scientific standard.

# Response

**Published:** 2022-10-13

**Authors:** Taufan Bramantoro

**Affiliations:** Airlangga University, Indonesia

Thank you for the valuable advices. The new version has been uploaded.
